# Probiotic-Derived Metabolites from *Lactiplantibacillus plantarum* OC01 Reprogram Tumor-Associated Macrophages to an Inflammatory Anti-Tumoral Phenotype: Impact on Colorectal Cancer Cell Proliferation and Migration

**DOI:** 10.3390/biomedicines13020339

**Published:** 2025-02-03

**Authors:** Beatrice Garavaglia, Letizia Vallino, Alessandra Ferraresi, Angela Amoruso, Marco Pane, Ciro Isidoro

**Affiliations:** 1 Laboratory of Molecular Pathology, Department of Health Sciences, Università del Piemonte Orientale, Via P. Solaroli 17, 28100 Novara, Italy; beatrice.garavaglia@uniupo.it (B.G.); letizia.vallino@uniupo.it (L.V.); alessandra.ferraresi@med.uniupo.it (A.F.); 2 Probiotical S.p.A., Via E. Mattei, 3, 28100 Novara, Italy; a.amoruso@probiotical.com (A.A.); m.pane@probiotical.com (M.P.)

**Keywords:** macrophage, probiotics, microbiota, inflammasome, tumor microenvironment

## Abstract

**Background:** Tumor-associated macrophages (TAMs) are key players in the colorectal cancer (CRC) tumor microenvironment (TME), representing the most abundant immune cells within it. The interplay between the intestinal microbiota, macrophages, and cancer cells significantly impacts tumor progression by driving macrophage polarization. Particularly, the polarization into the pro-tumoral M2-like TAM phenotype promotes the extracellular matrix remodeling, cancer cell proliferation, metastasis, immune suppression, and therapy resistance. Probiotic metabolites can disrupt this crosstalk, possibly reverting the TAM polarization toward a pro-inflammatory anti-tumoral phenotype, thus potentially benefiting the intestinal mucosa and opposing CRC progression. Previously, we showed that *Lactiplantibacillus plantarum* OC01 metabolites counter interleukin (IL)-6-induced CRC proliferation and migration. **Methods:** Here, we explore how probiotics affect CRC secretome and how this influences TAM polarization, which then impacts CRC malignancy. **Results:** The conditioning medium (CM) from CRC cells indeed promoted the polarization of macrophage toward the M2-like phenotype, whereas the CM from CRC pre-treated with *L. plantarum* OC01 metabolites induced a pro-inflammatory macrophage phenotype, characterized by NLRP3 inflammasome activation and reactive oxygen species (ROS) production, and by decreased expression of the M2 phenotype markers CD206 and CD163. Consistently, the expression of tumor growth factor (TGF)-β, a promoter of M2 macrophage polarization, was reduced in CRC cells treated with *L. plantarum* OC01. The pro-inflammatory macrophages inhibited CRC proliferation and migration. **Conclusions:** Overall, our study highlights the potential of metabolites from *L. plantarum* OC01 to reprogram the metabolism in cancer cells and thus reshape the TME by shifting TAMs toward a more inflammatory and anti-tumoral phenotype, emphasizing the promise of probiotics in advancing novel therapeutic approaches for CRC.

## 1. Introduction

Colorectal cancer (CRC) accounts for more than 900,000 deaths annually, making it the third most common cancer and the second leading cause of cancer-related deaths worldwide [1]. Its rising incidence has been linked to genetic and epigenetic factors, as well as dietary habits and sedentary lifestyles, particularly in developing countries [1]. In addition to these factors, many studies have demonstrated the significant role of the gut microbiota in CRC development and treatment outcomes [2,3]. Dysbiosis of the gut microbiota contributes to CRC through various mechanisms, including the secretion of microbial toxins, altered metabolites production, hormonal dysregulation, chronic immune activation, and persistent inflammation [4,5,6].

Given its crucial involvement in CRC pathogenesis, the gut microbiota is now recognized as a key component of the tumor microenvironment (TME) [7,8], which also includes stromal cells, immune cells, and non-cellular components such as extracellular matrix (ECM), cytokines, chemokines, and growth factors that support tumor growth [9]. Among immune cells, intestinal macrophages are the most abundant tumor-infiltrating immune cells in CRC tissues. These cells regulate the innate immune response, maintain tissue homeostasis, and modulate inflammation [10].

Within the TME, macrophages differentiate into tumor-associated macrophages (TAMs), exhibiting phenotypic plasticity [11]. TAMs can polarize into either an M1 or M2 phenotype, driven by signals released by cancer cells [12]. M1-like phenotype exhibits pro-inflammatory, immunostimulatory, and anti-tumorigenic properties through the release of inflammatory cytokines, such as interleukin (IL)-1β, IL-6, and tumor necrosis factor (TNF)-α. In contrast, the M2-like phenotype is characterized by immunosuppressive and pro-tumorigenic properties due to the secretion of IL-10, transforming growth factor (TGF)-β, and proteolytic enzymes like MMP-9 that contribute to ECM remodeling and facilitate cancer invasion and metastasis [11,13].

The polarization toward the M1-like phenotype is induced by microbial stimuli such as exogenous lipopolysaccharide (LPS) and other pro-inflammatory signals [14,15], whereas the M2-like phenotype can be divided into four distinct subtypes, namely M2a, M2b, M2c, and M2d, depending on the stimuli involved. M2a is induced by IL-4 or IL-13, M2b by immune complexes in combination with IL-1β or LPS, M2c by IL-10 and TGF-β, and M2d by toll-like receptors (TLR) or IL-6 signaling [16]. These subtypes can be identified by specific surface markers, with M1 macrophage expressing CD80 and CD86 on the membrane and M2c macrophages exhibiting elevated CD206 and CD163 expression [17].

M1 and M2 macrophages differ significantly in their immunological roles. M1 macrophages are activated through the NLRP3 inflammasome [18,19,20], which may be triggered by the production of reactive oxygen species (ROS) [21]). This activation leads to the release of pro-inflammatory cytokines IL-1β and IL-18 [22]. Furthermore, ROS can activate the NF-kB signaling pathway, enhancing the transcription of additional pro-inflammatory cytokines, playing a crucial role in inducing inflammatory responses and tumor cell eradication [17]. In contrast, M2 macrophages are characterized by the release of anti-inflammatory cytokines, having an immunosuppressive role and promoting tumor growth, angiogenesis, and metastasis.

During cancer progression, M1 and M2 macrophage phenotypes are not fixed but change over time. In the early stages of tumor development, M1 macrophages predominate and actively work to destroy cancer cells. As the tumor progresses, macrophages gradually adopt the M2 phenotype, which supports immune suppression and tumor growth [23]. Dysbiosis of the gut microbiota plays a key role in this process by activating macrophages toward M2-like phenotype, creating an immunosuppressive TME that facilitates tumor progression and metastasis [24].

Probiotics represent a promising new approach for managing CRC. By competing with pathogenic bacteria for space and nutrients and producing antibacterial substances, probiotics help restore a balanced gut microbiota (eubiosis). This balance exerts anti-tumor activity by counteracting carcinogenic molecules, promoting apoptosis and cellular differentiation, and regulating immune and inflammatory responses [25,26].

The objective of our study is to integrate our knowledge of the beneficial effects of probiotic metabolites into CRC management, focusing on the changes they induce in the TME. We hypothesize that metabolites produced by the gut microbiota may have the potential to disrupt the crosstalk between cancer and TAMs, whose polarization into M2-like phenotype contributes to cancer progression.

In our previous research, we investigated the effects of the supernatant from the probiotic strain *Lactiplantibacillus plantarum* OC01 on oncogenic pathways, which are commonly targeted in cancer treatment. Our findings revealed that metabolites derived from *L. plantarum* had a negative impact on the activation of ERK and S6, key pathways involved in cancer development. Furthermore, these probiotic-derived metabolites were able to counteract IL-6-induced cell proliferation and migration, exerting a beneficial effect on CRC cells [27].

Here, we demonstrate that *Lactiplantibacillus plantarum* OC01 metabolites modulate the TME by influencing the crosstalk between cancer cells and macrophage. Conditioning medium (CM) from probiotic-treated CRC cells induced in macrophage a shift toward a more pro-inflammatory phenotype characterized by activation of the NLRP3 inflammasome and increased ROS production. This shift was reflected in the decreased levels of TGF-β, a key cytokine involved in M2 polarization, and in probiotic-treated cancer cells, alongside not significant changes in pro-inflammatory cytokines such as IL-6, IL-1β, and TNF-α. The more inflammatory phenotype was further confirmed by a reduction in the expression of M2-associated markers CD206 and CD163 in PMA-differentiated THP-1 macrophages exposed to CM from probiotic-treated CRC cells, compared to those exposed to CM from untreated cells. In co-culture experiments, macrophages differentiated with CM from probiotic-treated cancer cells were able to decrease cancer cell proliferation and migration, whereas those differentiated with CM from untreated cells promoted these processes, highlighting that the shift toward a more pro-inflammatory macrophage phenotype is a feasible goal to combat the cancer. These findings suggest that probiotics alter the TME by modulating macrophage behavior and inflammatory cytokine profiles, ultimately influencing cancer cell progression.

By elucidating the impact of microbiota metabolites on TAMs and their role in modulating the pro-tumoral phenotype, our findings provide the pre-clinical rationale for probiotic therapeutic application, targeting both the gut microbiota and immune components of the TME. Restoring a healthy microbiota through probiotics is expected to improve the overall health status of CRC patients and potentially mitigate the risks of secondary metastasis and relapse.

## 2. Materials and Methods

### 2.1. Cell Culture and Treatment

The human THP-1 monocyte cell line (TIB-202™), isolated from the peripheral blood of an acute monocytic leukemia patient, and the human CRC cell lines HCT116 (CCL-247™) and HT29 (CCL-227™), each characterized by distinct genetic backgrounds and tumor site origins, were obtained from the American Type Culture Collection (ATCC). All cell lines were cultured in RPMI-1640 medium (R8758; Sigma-Aldrich, St. Louis, MO, USA) supplemented with 10% heat-inactivated fetal bovine serum (FBS, ECS0180L; Euroclone, Milan, Italy), 1% glutamine (G7513; Sigma-Aldrich, St. Louis, MO, USA), and 1% penicillin/streptomycin (PES, P0781; Sigma-Aldrich, St. Louis, MO, USA). The cells were maintained under standard culture conditions (37 °C, 95% air, 5% CO_2_).

THP-1 monocytes were treated with 20 ng/mL Phorbol-12-Myristate-13-Acetate (PMA, P8139; Sigma-Aldrich, St. Louis, MO, USA) and dissolved in DMSO to induce their differentiation into macrophages.

### 2.2. Probiotic Formulation

The supernatant from *Lactiplantibacillus plantarum* OC01 (NCIMB 30624), sourced from the Probiotical SpA collection, was prepared by culturing the bacteria as previously described [27].

For stimulation experiments, eukaryotic cells were exposed to the cell-free supernatant of OC01 after filtering the bacteria culture broth through a 0.2 μm syringe filter. This supernatant contains all catabolic and anabolic products released by 10 billion viable probiotic cells after overnight overgrowth.

CRC cultures were supplemented with 10 μL of OC01 cell-free supernatant per 1 mL of final culture media volume.

### 2.3. Collection of the Colorectal Cancer Cell Conditioning Medium (CRC Cell CM)

CRC cells (HCT116 and HT29) were cultured in 75 cm^2^ flasks and treated with or without *Lactiplantibacillus plantarum* OC01 supernatant for 48 h, and then the CM was collected. CRC cell CM was centrifuged at 2000 rpm for 5 min to remove cell debris, diluted 50:50 (CRC cell CM: RPMI-1640), and then stored at −20 °C until use, as previously described [28].

### 2.4. Antibodies

The following primary antibodies (at the dilution indicated) were used for either immunofluorescence or Western blotting: mouse anti-CD68 (1:500, cod. 14-0688-80; Life Technologies, Paisley, UK); rabbit anti-NLRP3 (1:500, cod. MA5-32255; Life Technologies, Paisley, UK); rabbit anti-CD206 (1:500, cod. MA5-32498; Life Technologies, Paisley, UK); mouse anti-CD163 (1:500, cod. ab156769; abcam, Cambridge, UK); mouse anti-p21 (1:100, cod. B1313; Santa Cruz, Biotechnology, Dallas, TX, USA); rabbit anti-Ki67 (1:100, cod. HPA001164; Sigma-Aldrich, St. Louis, MO, USA); rabbit anti-GAPDH (1:1000, cod. G9545; Sigma-Aldrich, St. Louis, MO, USA); and mouse anti-β-actin (1:2000, cod. A5441; Sigma-Aldrich, St. Louis, MO, USA).

### 2.5. Western Blotting Analysis

THP-1 cells were seeded (50,000 cells/cm^2^) in p35 Petri dishes and treated as indicated. Cell homogenates were prepared by freeze–thawing and ultrasonication in RIPA lysis buffer containing protease inhibitors. Equal protein amounts (30 μg) were separated by SDS-PAGE and transferred to PVDF membranes. Membranes were incubated overnight with primary antibodies at 4°C, followed by incubation with secondary HRP-conjugated antibodies for 1 h at room temperature, as detailed in [29]. Bands were detected using Enhanced Chemiluminescence reagents (cod. NEL105001EA; Perkin Elmer, Waltham, MA, USA) and analyzed using a VersaDOC Imaging System (BioRad, Hercules, CA, USA) with Quantity One software (v.4.5). Band intensity was quantified by densitometry using Quantity One software (v.4.5), and normalization was performed by re-probing the membranes with GAPDH or β-actin. Data were reproduced at least three times.

### 2.6. Immunofluorescence Assay

Cells were seeded onto sterile coverslips at a density of 30,000 (cancer cells) – 50,000 (THP-1) cells/cm^2^ and treated as reported. At the end of the experiment, the coverslips were fixed in ice-cold methanol (cancer cells) or 4% paraformaldehyde (THP-1) and incubated overnight at 4 °C with specific primary antibodies as previously described [29]. The following day, coverslips were incubated for 1 h at room temperature with secondary antibodies (diluted in 0.1% Triton-PBS + 10% FBS). AlexaFluor488-conjugated goat-anti rabbit IgG (1:000, cod. A32731, Invitrogen, Paisley, UK) or AlexaFluor555-conjugated goat-anti mouse IgG (1:1000, cod. A32727, Invitrogen, Paisley, UK) was used, as appropriate. Nuclei were stained with UV fluorescent dye DAPI (4′,6-diamidino-2-phenylindole). Coverslips were mounted onto glass using SlowFade reagent (cod. S36936; Life Technologies, Paisley, UK) and imaged with a fluorescence microscope (Leica Microsystems DMI6000; Wetzlar, Germany).

### 2.7. Phagocytosis Study

THP-1 cells were plated onto sterile coverslips at a density of 50,000 cells/cm^2^ and treated as described. To evaluate phagocytic activity, cells were first incubated with 100 nM LysoTracker™ Red probe (cod. L7528, Life Technologies, Paisley, UK) at 37 °C for 10 min, followed by incubation with 25 µg/mL of 30 nm carboxy-functionalized nanoparticles (COOH-NPs), emitting green fluorescence (cod. L5155, Sigma-Aldrich, St. Louis, MO, USA), at 37 °C for 10 min [30]. Coverslips were washed, mounted, and immediately imaged using a fluorescence microscope (Leica Microsystems DMI6000, Wetzlar, Germany).

### 2.8. Anion Superoxide Production by MitoSOX™ Fluorescence Assay

THP-1 cells were seeded onto coverslips at a density of 50,000 cells/cm^2^ and treated as described. Mitochondrial anion superoxide was detected in living cells using 5 μM MitoSOX™ Red (cod. M36008; Life Technologies, Paisley, UK) as previously described [31]. Coverslips were washed three times with PBS, mounted on glass slides, and images were immediately acquired using a fluorescence microscope (Leica Microsystems DMI6000, Wetzlar, Germany).

### 2.9. RNA Isolation and Quantitative PCR

HCT116 and HT29 cells were plated in p60 Petri dishes at a density of 50,000 cells/cm^2^ and treated as described. The total RNA was extracted from the cells using TRIzol reagent (cod. T9424, Sigma-Aldrich, St. Louis, MO, USA). The mRNA was then reverse-transcribed into complementary DNA (cDNA) using the RevertAid First Strand cDNA Synthesis Kit (cod. K1622, Thermo-Scientific, Waltham, MA, USA). The cDNA was then amplified by PCR in the presence of recombinant Taq DNA polymerase (cod. 10342-020, Invitrogen, Waltham, MA, USA) and primers designed to anneal to the 5′- and 3′-untranslated regions (UTRs). The PCR products ware analyzed by agarose gel electrophoresis.

The sequences of the primers used were as follows (Table 1):

### 2.10. Macrophages and Colorectal Cancer Cell Lines Co-Culture System Assay

To partially recreate the in vivo interaction between cancer and TAMs, a non-contact co-culture system (Transwell^®^ Permeable Supports, Polycarbonate Membrane, 12 mm Insert, 0.4 μm pore size; cod. 3413, Corning Incorporated Costar, NY, USA) was used to assess the proliferative capacity of cancer cells exposed to the presence of macrophages. THP-1 cells were plated at a density of 30,000 cells/cm^2^ within the insert, differentiated into macrophages as reported, and subsequently transferred to a 24-well plate with CRC cells pre-seeded on coverslips. After 24 h of co-culture, the coverslips with cancer cells were washed with PBS, fixed with ice-cold methanol, and permeabilized with 0.2% Triton X-100 in PBS. The cells were then incubated with primary antibodies and processed for immunofluorescence staining as described above.

To assess the migration capability of cancer cells in co-culture with THP-1-differentiated macrophages, THP-1 cells were seeded into a 24-well plate at a density of 50,000 cells/cm^2^ and differentiated into macrophages as described. Cancer cells were seeded in p35 Petri dishes at a density of 40,000 cells/cm^2^ and cultured until they reached 80% confluence. After 48 h of culture, the cancer cells were trypsinized, collected, and counted. An aliquot of 50,000 cells per experimental condition was resuspended in serum-free medium and plated into uncoated inserts (Transwell^®^ Permeable Supports, Polycarbonate Membrane; 6.5 mm Insert; 8.0 μm pore size Polycarbonate Membrane; cod. 3422, Corning Incorporated Costar, NY, USA). Each insert was placed onto THP-1-differentiated macrophages in a 24-well plate containing complete RPMI, and the plate was placed in the incubator. After 24 h of incubation, a fraction of each cell population had migrated through the porous membrane to the underside of the inserts. The migrated cells were washed in PBS, fixed in methanol for 30 min, washed again in PBS, and then stained for 1 h with eosin–hematoxylin solution (cod. 05-M06002; Bio-Optica, Milan, Italy). The inserts were washed in PBS, left to dry for at least 1 day, then cut and mounted onto slides using SlowFade reagent (cod. S36936; Life Technologies, Paisley, UK). Photographs were taken from random fields using an Axioscan 7 microscope (magnification 20X; ZEISS, Oberkochen, Germany). The amount of migrating cells (reflected by staining intensity) was quantified using ImageJ software. Data are expressed as the average number of migrated cells from different fields per each condition.

### 2.11. Statistical Analysis

All data refer to at least three separate experiments. Data in histograms are shown as average ± S.D. Statistical analysis was performed with GraphPad Prism 5.0 software. Bonferroni’s multiple comparison test after one-way ANOVA analysis (unpaired, two-tailed) and t student test were employed. Significance was considered as follows: **** *p* < 0.0001; *** *p* < 0.001; ** *p* < 0.01; * *p* < 0.05.

## 3. Results

### 3.1. Differentiation of THP-1 into Macrophage-like Phenotype

**Figure 1:**
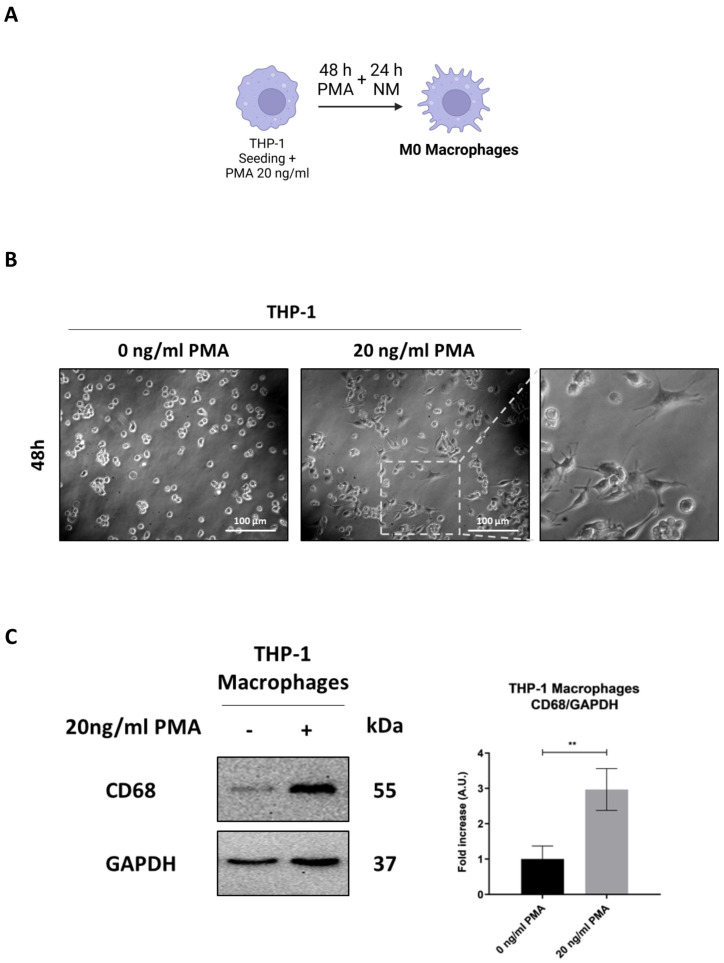

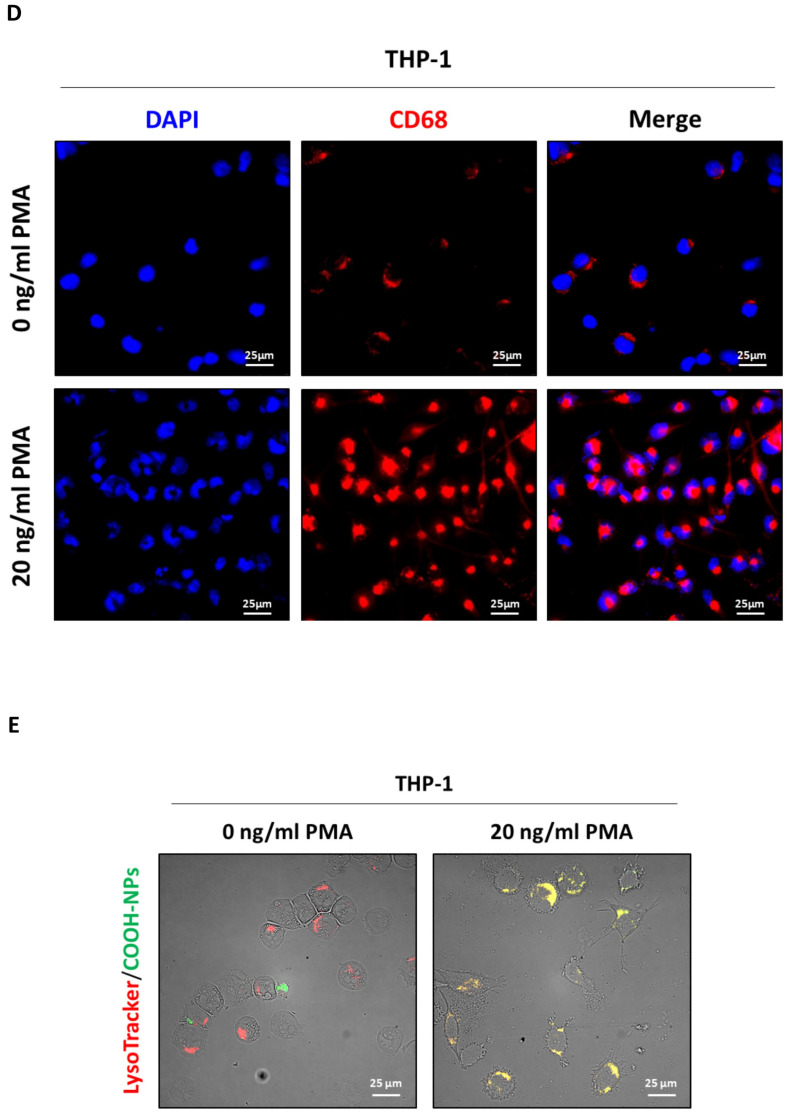
THP-1 cell differentiation into M0 macrophage. (**A**) Schematic representation of experimental design. THP-1 cells were plated and treated with or without 20 ng/mL of PMA for 48 h, followed by a one-day recovery in normal media (NM) (created with BioRender). (**B**) Cell morphology images of THP-1 were obtained using phase-contrast microscopy (scale bar = 100 μm, magnification = 20×). (**C**) Cell homogenates were analyzed by Western blotting for the expression of the M0 macrophage marker CD68. The filter was re-probed with GAPDH as a loading control. The blots are representative of three experiments with reproducible results. Densitometric analysis, including standard deviation, is provided (significance was considered as ** *p* < 0.01). (**D**) THP-1 cells were plated on coverslips and treated as described. At the end of the treatment, cells were stained for the M0 macrophage marker CD68 (red) and imaged using fluorescence microscopy (scale bar = 25 μm, magnification = 63×). Nuclei were stained with DAPI. Representative images for each condition are shown. (**E**) THP-1 cells were plated onto coverslips and treated as described. At the end of the treatment, cells were incubated with Lysotracker™ probe for 10 min at 37 °C and then exposed to 25 µg/mL COOH-NPs for an additional 10 min to evaluate their internalization into lysosomes (yellow signal). Images were acquired using fluorescence microscopy (scale bar = 25 μm, magnification = 63×). Representative images for each condition are shown.

The human monocyte cell line THP-1, commonly used for studies on macrophage differentiation, was exposed or not to 20 ng/mL of Phorbol-12-Myristate-13-Acetate (PMA) to stimulate differentiation into macrophages (Figure 1A). Following 48 h of incubation with PMA and a subsequent 24 h recovery in normal media, treated cells exhibited increased adherence to the culture plate, a key indicator of macrophage differentiation, along with a stellate and polygonal morphology and extended pseudopodia. In contrast, control cells retained a rounded shape and remained in suspension (Figure 1B). This phenotypic transition was confirmed by monitoring the expression of the pan-macrophage or M0-like phenotype marker CD68 through Western blotting and immunofluorescence. The significant increased expression of CD68 in PMA-treated cells confirmed the activation of THP-1 monocytes into M0-like phenotype macrophages (Figure 1C,D).

CD68, a transmembrane glycoprotein primarily localized in lysosomes, plays a crucial role in the internalization and degradation of extracellular particles, facilitating phagocytosis. Its upregulation is associated with enhanced macrophage activity [32]. To assess the phagocytic capacity of M0 macrophages, cells were incubated with fluorescence-tagged nanoparticles (NPs), and their uptake into lysosomes was visualized using a red LysoTracker™ probe. Co-localization of NPs and lysosomes (yellow signal) indicated successful phagocytosis of NPs by macrophages. PMA-treated cells showed increased co-localization, suggesting enhanced phagocytic activity, whereas in untreated cells, NP uptake into lysosomes was completely absent, confirming the lack of phagocytic function in the monocyte cells (Figure 1E).

### 3.2. Conditioning Medium from Lactiplantibacillus plantarum OC01-Treated Colorectal Cancer Cells Promotes Macrophage Inflammatory Phenotypes via NLRP3 Activation

Macrophages within the tumor microenvironment (TME) are strongly influenced by cytokines, chemokines, and growth factors, particularly secreted by cancer cells, driving their polarization into either a classically activated M1-like phenotype or an alternatively activated M2-like phenotypes. These tumor-associated macrophages (TAMs) play a critical role in colorectal cancer (CRC) progression and metastasis [33].

Given the pivotal role of TAMs in mediating the inflammatory response within the TME, we investigated inflammasome activation, focusing on NLRP3 as the central component in inflammasome-driven inflammation [34]. M1 macrophages, associated with a pro-inflammatory and anti-tumor phenotype, typically exhibit higher levels of NLRP3 activity, while M2 macrophages are characterized by reduced NLRP3 activity, consistent with their anti-inflammatory profile and tumor-promoting effects [19]. Notably, mitochondrial reactive oxygen species (ROS) have been identified as an inducer of NLRP3 activation, linking oxidative stress to inflammasome activation and the inflammatory response [21].

**Figure 2:**
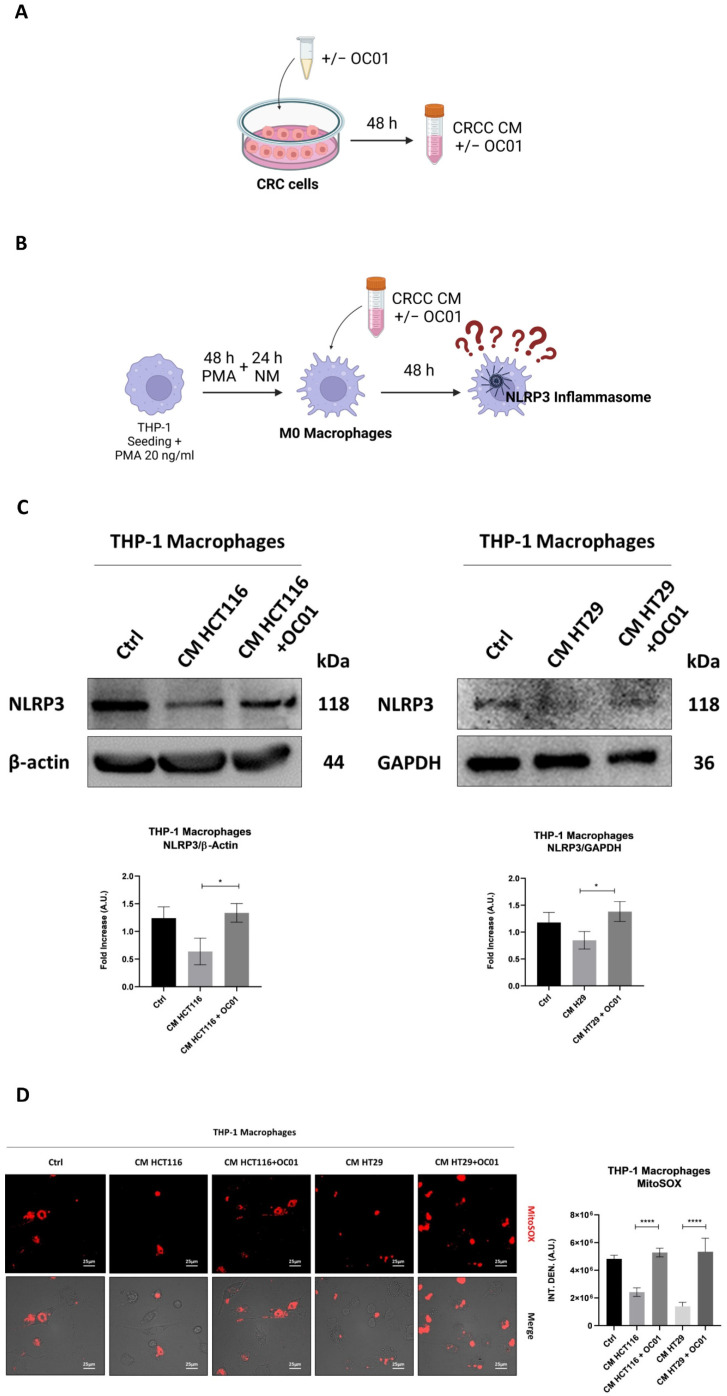
The secretome of the *Lp*OC01-SN-cured colorectal cancer cell induces NLRP3 activation and ROS generation in macrophages. (**A**,**B**) Schematic representation of experimental design. HCT116 and HT29 cells were plated and treated or not with 10 µL of *Lp*OC01-SN for 48 h. The treatment was renewed every 24 h. At the end of the treatment period, the CM was collected and used to treat the differentiated M0 macrophages for 48 h (created with BioRender). (**C**) Cell homogenates were analyzed by Western blotting to assess the expression of NLRP3. The blot is representative of three experiments with reproducible results. The filter was re-probed with β-actin or GAPDH as a loading control. Densitometric analysis, including standard deviation, is provided (significance was considered as * *p* < 0.05). (**D**) THP-1 cells were plated on coverslips and differentiated and treated as described above. After 48 h of treatment, cells were labeled with MitoSOX™. Coverslips were directly visualized using a fluorescence microscope (scale bar = 25 μm, magnification = 63×). The data presented are representative of multiple fields for each condition. Graphs report the quantification of the fluorescence intensity per cell (significance was considered as **** *p* < 0.0001).

To investigate the effect of CRC cells secretome on NLRP3 activation in macrophages, differentiated M0 macrophages were exposed to the conditioning medium (CM) from two CRC cell lines, HT29 and HCT116, which have different genetic backgrounds. The cancer cells were pre-treated for 48 h with or without *Lactiplantibacillus plantarum* OC01 supernatant (*Lp*OC01-SN) to determine whether this treatment alters their secretion that would then influence the inflammatory status of macrophages (Figure 2A,B).

Macrophages incubated with CM from HCT116 and HT29 cells pre-treated with *Lp*OC01 metabolites exhibited elevated NLRP3 expression and increased ROS production. In contrast, macrophages incubated with CM from untreated cancer cells showed reduced NLRP3 expression and minimal ROS production (Figure 2C,D). These findings suggest that macrophages exposed to CM from *Lp*OC01-SN-cured cancer cells may adopt a more inflammatory phenotype, potentially indicative of an M1-like profile. Conversely, macrophages exposed to CM from untreated cancer cells appear to exhibit a less inflammatory status, potentially aligning with an M2-like profile.

### 3.3. Lactiplantibacillus plantarum OC01 Metabolites Reduce the Expression of TGF-β in Colorectal Cancer Cells

Cytokines in the TME can direct macrophage activation toward an anti-inflammatory, pro-tumoral phenotype, or toward a pro-inflammatory, anti-tumoral phenotype, thus influencing the overall immune landscape within the tumor. Cancer cells significantly contribute to an immune-suppressive, pro-tumoral environment, particularly through the secretion of cytokines like IL-10, TGF-β, and colony-stimulating factor-1 (CSF-1). These molecules help reprogram macrophages to adopt an M2-like phenotype, promoting tumor progression by suppressing immune surveillance and enhancing tissue remodeling [35,36,37,38]. In contrast, cytokines, such as TNF-α, are generally associated with a pro-inflammatory, tumor-suppressive macrophage phenotype [39], which can enhance the ability of the immune system to fight against the tumor.

To investigate the mechanism by which *Lp*OC01 modulates CRC cell secretions and, thereafter, macrophage inflammatory status, we analyzed the cytokine profile of HCT116 and HT29 cells. We specifically analyzed key cytokines such as IL-8, IL-1β, IL-10, IL-18, IL-6, TNF-α, and TGF-β that are known to play pivotal roles in shaping the TME.

**Figure 3:**
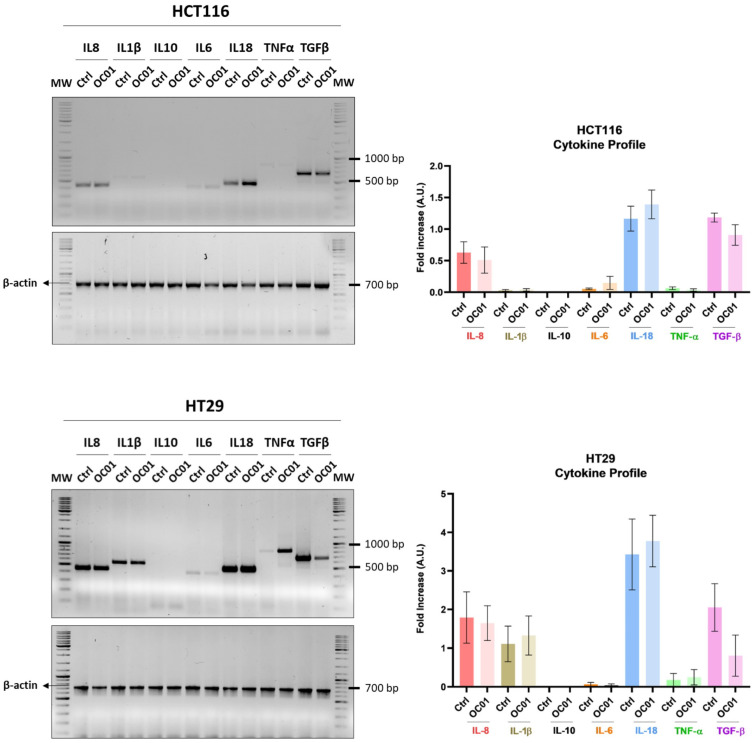
*Lp*OC01 cell-free supernatant reduces TGF-β expression in HCT116 and HT29 cells. Cells were seeded in Petri dishes and treated with or without 10 µL of *Lp*OC01-SN for 48 h, with medium and treatment renewed every day. Agarose gel electrophoresis was used to visualize the PCR products for IL-8, IL-1β, IL-10, IL-18, IL-6, TNF-α, and TGF-β. The cytokine expression profiles shown are representative of three experiments with reproducible results.

Our results demonstrated that treatment of CRC cells with *Lp*OC01-SN led to a marked reduction in TGF-β expression, particularly in HT29 cells. Additionally, a slight though not significant change in IL-1β, IL-6, and TNF-α levels was observed in both HCT116 and HT29 cells (Figure 3).

Our results suggest that metabolites from *Lactiplantibacillus plantarum* OC01 may alter the cytokine profile of cancer cells, creating an environment that promotes a more pro-inflammatory macrophage state, potentially limiting tumor progression.

### 3.4. Conditioning Medium from Lactiplantibacillus plantarum OC01-Treated Colorectal Cancer Cells Reduces the Polarization of Macrophages into the M2-like Phenotype

To further investigate whether the observed changes in inflammasome activation in macrophages exposed to CM from *Lp*OC01-SN-cured cancer cells were linked to a shift in macrophage phenotype, we assessed the expression of the M2 markers CD206 and CD163, known to be associated with a less inflammatory state and pro-tumorigenic activity [40].

**Figure 4:**
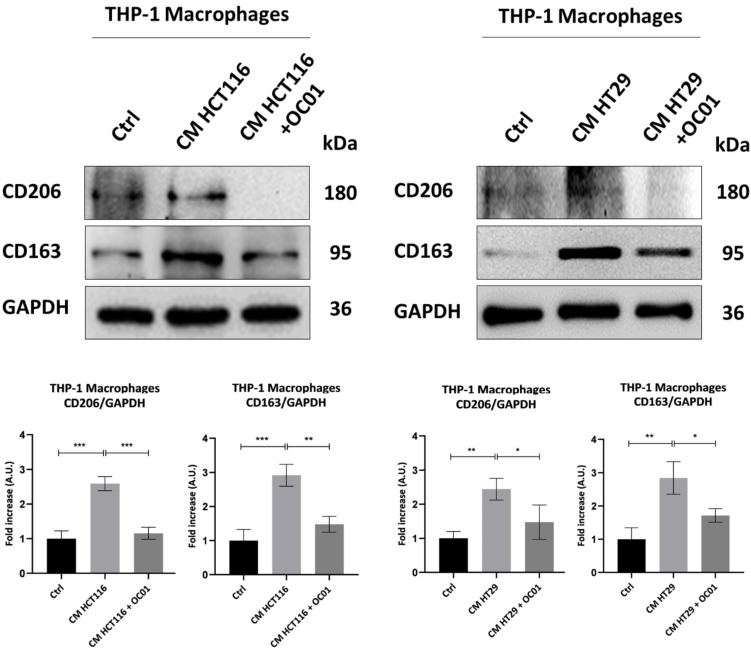
CM from *Lp*OC01-SN-cured CRC cells limits the differentiation into M2 macrophages. HCT116 and HT29 cells were plated and treated or not with 10 µL of *Lp*OC01-SN for 48 h. The treatment was renewed every 24 h. At the end of the treatment period, the CM was collected and used to treat the differentiated M0 macrophages for 48 h. Cell homogenates were analyzed by Western blotting to assess the expression of M2 macrophages markers CD206 and CD163. The filter was re-probed with GAPDH as a loading control. The blot is representative of three experiments with reproducible results. Densitometric analysis, including standard deviation, is provided (significance was considered as follows: *** *p* < 0.001; ** *p* < 0.01; * *p* < 0.05).

Our results showed that CM from CRC cells supplemented with *Lp*OC01-SN significantly reduced CD206 and CD163 expression, thereby limiting the differentiation of M0 macrophages into an M2-like phenotype. In contrast, macrophages exposed to CM from untreated CRC cells displayed an increased expression of these markers, indicative of differentiation into anti-inflammatory M2 macrophages, consistent with reduced inflammasome activation. Conversely, the reduction in CD206 and CD163 expression combined with increased NLRP3 expression and ROS production in macrophages treated with CM from *Lp*OC01-SN-cured CRC cells suggests a shift toward a pro-inflammatory macrophage state (Figure 4).

### 3.5. Macrophages Reprogrammed by Conditioning Medium from LpOC01-SN-Cured CRC Cells

We have demonstrated that the CM of cancer cells promotes the differentiation of macrophages toward an M2-like phenotype, associated with anti-inflammatory properties. In contrast, the CM of cancer cells exposed to *Lp*OC01-SN induces a more inflammatory macrophage phenotype that does not express M2-like markers.

**Figure 5:**
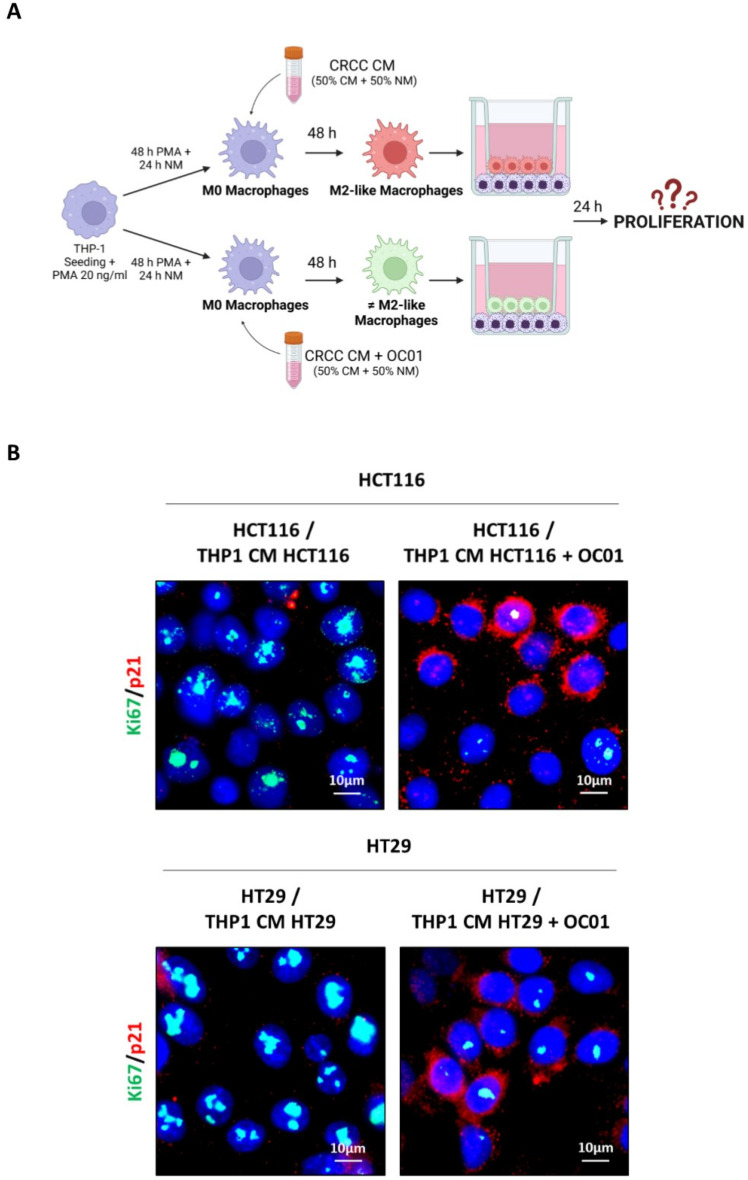

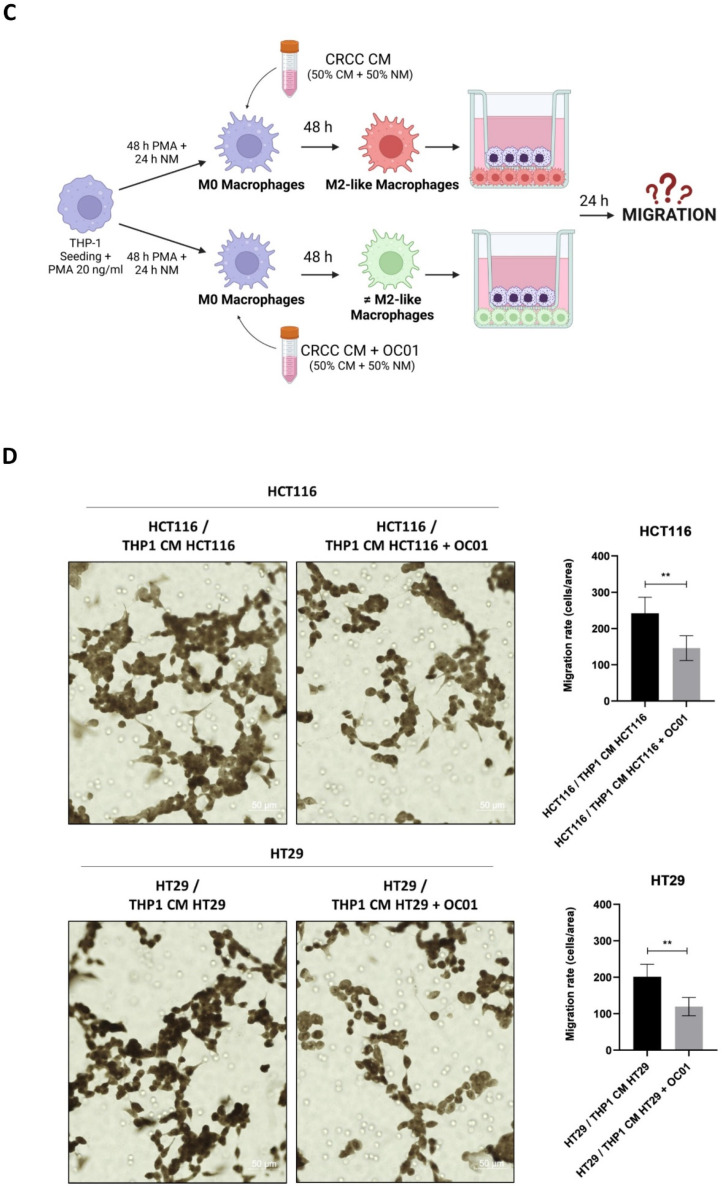
Macrophages reprogrammed by the conditioning medium from *Lp*OC01-SN-cured CRC cells reduce the proliferation and migration of HCT116 and HT29 cells. (**A**) Schematic representation of experimental design. THP-1 cells were plated on Transwell inserts (upper chamber) and differentiated into macrophages (48 h with PMA plus 24 h in normal medium and further incubated for 48 h with the CM of CRC cells pre-treated or not with 10 µL of *Lp*OC01-SN). The inserts were then transferred into a 24-well plate pre-seeded with CRC cells on coverslips for 24 h (created with BioRender). (**B**) Following 24 h of co-culture in normal (complete) medium, the HCT116 and HT29 cells on the coverslip were processed for immunofluorescence staining of Ki67 (green) and p21 (red). Nuclei were stained with DAPI. The cells were photographed under fluorescence microscopy (scale bar = 10 μm, magnification = 63×). Representative images for each condition are shown. (**C**) Schematic representation of experimental design. THP-1 cells were plated on the lower chamber and differentiated into macrophages (48 h with PMA plus 24 h in normal medium and further incubated for 48 h with the CM of CRC cells pre-treated or not with 10 µL of *Lp*OC01-SN). HCT116 and HT29 cells were plated into Transwell inserts which were then positioned in a 24-well plate. The upper chamber containing CRC cells was filled with serum-free medium (to avoid possible influence by serum factors), while the lower chamber containing the macrophages was filled with normal (complete) medium. Incubation lasted 24 h (created with BioRender). (**D**) After 24 h of co-culture, migrated HCT116 and HT29 cells were imaged in random fields using an Axioscan 7 microscope (scale bar = 50 µm, magnification = 20×). Representative images for each condition are shown, along with a quantification of migrated cells, presented with standard deviation (statistical significance: ** *p* < 0.01).

To investigate how these differentially polarized macrophages impact the behavior of HCT116 and HT29 cancer cells, a non-contact co-culture system using Transwell inserts was employed. This system allowed the exchange of soluble factors without direct cell–cell contact. Differentiated macrophages (M0 macrophages exposed to the CM from HCT116 or HT29 cells that had been or not exposed to *Lp*OC01-SN for 48 h) were seeded in the upper compartment of the Transwell system, while cancer cells were plated in the lower compartment (Figure 5A). After 24 h of co-culture, the proliferation of cancer cells was analyzed by immunofluorescence assessment of the expression of the proliferative marker Ki67 and of the cyclin-dependent kinase inhibitor p21waf/Cip1 by. As shown in Figure 5B, co-culture with M2-like macrophages resulted in increased Ki67 expression and decreased p21waf/Cip1 in HCT116 and HT29 cells. In contrast, when cancer cells were co-cultured with more inflammatory macrophages differentiated with CM from cancer cells pre-cured with *Lp*OC01-SN, a decrease in cancer cell proliferation was observed, along with an increase in p21waf/Cip1 and a reduction in Ki67 expression.

The impact of different macrophage phenotypes on cancer cell migration was further investigated. In this setup, cancer cells were plated in the upper compartment, while macrophages differentiated as described above were seeded in the lower compartment of the Transwell system (Figure 5C). After 24 h, the number of cancer cells that migrated through the porous membrane was quantified. Co-culture with inflammatory macrophages differentiated with the CM from cancer cells pre-cured with *Lp*OC01-SN significantly reduced the migration of HCT116 and HT29 cells compared to co-culture with M2 macrophages obtained from incubation with the CM of untreated CRC cells (Figure 5D).

These results suggest that M2-like macrophages, which are more likely to promote a pro-tumoral environment, enhance cancer cell proliferation and migration, while macrophages with a more inflammatory phenotype inhibit these processes.

## 4. Discussion

The tumor mass exists within a complex microenvironment that includes cancer cells as well as stromal components such as fibroblasts, endothelial cells, immune cells, and adipocytes. These stromal cells engage in active communication with cancer cells through the exchange of soluble factors, including cytokines, chemokines, and growth factors, establishing bidirectional interactions. This dynamic crosstalk can drive stromal cells to adopt more aggressive and malignant phenotypes, contributing to tumor growth. As a result, the tumor microenvironment (TME) is increasingly recognized as a critical player in tumor initiation, progression, and metastasis [9].

In colorectal cancer (CRC), intestinal macrophages are the predominant immune cells infiltrating the tumor [41]. In their resting state, these macrophages exhibit an unpolarized M0-like phenotype [42]. However, signals from the TME, particularly those secreted by cancer cells, influence macrophage polarization, promoting them toward either a classically activated M1-like phenotype or an alternatively activated M2-like phenotype [15]. Additionally, the gut microbiota closely interacts with macrophages, shaping their behavior within the TME, further influencing tumor growth [3]. The interplay between immune cells, microbiota, and cancer cells significantly affect the development and progression of CRC [43].

Here, we investigated the effect of the *Lactiplantibacillus plantarum* OC01 supernatant (*Lp*OC01-SN) on the secretome of CRC cells and how this secretome would impact macrophage polarization. Understanding how these probiotic metabolites alter the secretome is crucial, as these modifications can significantly affect the TME, potentially slowing tumor growth and dissemination. Of note, one of the key processes influenced by the cancer secretome is the polarization of macrophages into tumor-associated macrophages (TAMs).

In the TME, TAMs predominantly adopt an M2-like phenotype. This polarization is driven by the abundance of anti-inflammatory cytokines, such as IL-4 and IL-10, which promote macrophage conversion into the M2 phenotype. Additionally, the metabolic landscape of the TME plays a significant role, with lactate being a driver of M2 phenoconversion [44]. M1 and M2 differ also in terms of energetic metabolism, with the former showing predominantly aerobic glycolytic metabolism and low production of ATP (thus relying on the availability of a huge amount of glucose) while the latter exploits at best the mitochondrial respiration [42]. Cancer cells predominantly utilize glucose through aerobic glycolysis to support their rapid proliferation, thereby limiting glucose availability for other cells. Consequently, macrophages within the TME lack glucose and rely on oxidative phosphorylation for energy production, which supports their M2 polarization. Additionally, cancer cells release lactic acid in the TME that further contributes to maintaining the M2 phenotype. Together, these conditions create an immunosuppressive environment that facilitates tumor progression and immune evasion [17].

Our study assessed the expression of NLRP3 to evaluate whether the macrophages polarized by CRC cells’ conditioning medium (CM) exhibited a pro-inflammatory or anti-inflammatory phenotype. Our results demonstrated that treating M0 macrophages with HCT116 and HT29 CM significantly reduced the levels of both NLRP3 and reactive oxygen species (ROS). However, when the macrophages were incubated with the CM from cancer cells that had been pre-treated with *Lp*OC01-SN, we observed a marked increase in NLRP3 and ROS levels. These findings suggested a differential inflammatory state, with a shift toward a more inflammatory phenotype in the latter scenario. This shift in macrophage inflammatory status appeared to be closely linked to alterations in the TME, particularly in the secretome of CRC cells. We have previously shown that *Lp*OC01-SN could contrast the growth of CRC cells, both in 2D- and 3D-spheroid cultures, along with the induction of autophagy [27]. It is likely that the (48 h) exposure to the *Lp*OC01-SN downregulates the glucose metabolism and alters the proteome in CRC cells so that their conditioned medium is poor in lactic acid and still rich in glucose. Such conditioned medium is then able to impact the metabolism of the macrophages shifting their polarization into an M1-like phenotype characterized by aerobic glycolysis and consequent mitochondrial dysfunctioning, with the production of ROS and activation of the inflammasome.

To define the polarized phenotype, we analyzed the expression of CD206 and CD163, surface markers characteristic of M2c macrophages [17]. These markers were significantly elevated when macrophages were incubated with CM from untreated cancer cells, indicating a pronounced M2c polarization. Conversely, the incubation with the CM from *Lp*OC01-SN precured CRC cells led to a notable decrease in CD206 and CD163 expression in macrophages, supporting that *Lactiplantibacillus plantarum* OC01 metabolites modified the TME by altering the cancer cell secretome. The M2c macrophages are known to be strongly induced by TGF-β [16,17]. Notably, the treatment with *Lp*OC01-SN induced a reduction in TGF-β (not other cytokines) levels in both HCT116 and HT29 cells. This decrease in TGF-β likely played a pivotal role in modulating macrophage polarization, specifically reducing the M2-like phenotype.

In summary, these findings underscore the capacity of the CRC cell secretome to induce an M2c-like phenotype in macrophages, driven in part by TGF-β. Precuring cancer cells with the probiotic reduced TGF-β levels and mitigated the M2c polarization, highlighting a critical interplay between the secretome of cancer cells and macrophage behavior. It should be noted that in our experimental setting, we attempted to reproduce in vitro the spatial relationship between the probiotics (which are administered orally and therefore are in contact with the epithelial mucosa) and the tumor cells that protrude into the lumen (and therefore are in contact with the intestinal microbiota and metabolites) while they also grow in the deeper layer of the extracellular matrix where they come into contact with stromal cells, including macrophages.

The anti-inflammatory phenotype of M2-like macrophages, particularly M2c, is widely associated with tumor-promoting activities, whereas a more inflammatory phenotype is linked to anti-tumor functions. By mimicking the in vivo interactions between cancer cells and TAMs, we further investigated these functional differences, establishng a co-culture system using transwells. This approach allowed us to evaluate how macrophages with distinct polarization states and inflammatory status impact tumor progression. In agreement with our previous findings, macrophages exhibiting an M2c-like phenotype, induced by the CM from untreated cancer cells, promoted cancer cell proliferation, as indicated by the reduced expression of p21 and increased expression of Ki67, and cancer cell migration, as indicated by the greater number of cells that migrated through the transwell. Conversely, macrophages with a more inflammatory phenotype, induced by CM derived from *Lactiplantibacillus plantarum* OC01-pre-treated CRC cells, displayed the opposite effect on cancer cells repressing cell proliferation, as indicated by the increased expression of p21 and decreased expression of Ki67, and cell migration. Macrophages are known to change their phenotype (and metabolism) quite rapidly to adapt the TME conditions, depending on the availability of nutrients, metabolites and cytokines (e.g., glucose, lactate, TGFβ, etc.). In our co-culture system, the macrophages maintained their activity during the 24 h of incubation in normal medium, suggesting that they could retain the acquired phenotype even in the absence of CRC secretion. This was confirmed with the CM from HT-29 cells, where the changes in TGFβ were more pronounced, by assessing the expression of specific macrophage subtype markers (Figure S1). However, we did not test whether the acquired phenotype of the macrophage lasts for a longer time in the absence of the external stimuli. Another limitation of the present study is that we have not yet identified the bioactive metabolites in the *Lp*OC01 supernatant responsbile for such beneficial effects, though likely butyrate is one of the main candidates as suggested by our previous work [27,29]

Overall, our results suggest that *Lactiplantibacillus plantarum* OC01 metabolites may modulate the cytokine secretion pattern of CRC cells, potentially driving macrophages toward a more pro-inflammatory state. This modulation could counteract the immune-suppressive signaling within the TME, offering a promising approach to altering macrophage polarization. Here, we have shown that macrophages polarized toward a pro-inflammatory phenotype contrast the growth and migration of CRC cells. The reduced secretion of TGFβ and of lactate by *Lp*OC01-SN-precured CRC cells is likely responsible for the shift toward a more inflammatory macrophage profile, potentially aligning with an M1-like phenotype or an intermediate phenotype between M1 and M2, such as M2b. M2b macrophages are known to be induced by IL-1β or LPS and express markers such as IL-10, IL-1 receptor, TNF-α, and M1 marker CD86 [45]. These macrophages are typically associated with a more balanced immune response, favoring a more pro-inflammatory environment within the TME. However, further investigation is required to definitively classify this phenotype and elucidate the precise mechanisms involved in macrophage polarization and activation.

In conclusion, this study highlights the potential of probiotic metabolites as an adjuvant therapy for CRC. By focusing on their ability to influence the TME, specifically through modulating the polarization and activation of TAMs, we aimed to uncover novel strategies to counteract immune suppression. Considering the critical role of the gut microbiota in CRC progression and treatment outcomes, these findings provide valuable insights into how probiotics can restore eubiosis, suppress tumor growth, and enhance the efficacy of conventional therapies. We propose the continuous administration of probiotics as adjuvant therapy in CRC to ensure that beneficial effects on the TME are maintained. Ultimately, this research contributes to the development of more effective, personalized approaches to CRC management by addressing both the microbiota and immune components of the TME.

## Figures and Tables

**Table 1:** Overview of the cytokines analyzed, including their amplicon lengths and the sequences of the forward and reverse primers used for amplification.

Primer	Amplicon Length	Forward 5′ -> 3′	Reverse 3′ -> 5′
IL-8	455 bp	GGACAAGAGCCAGGAAGAAA	CCTACAACAGACCCACACAATA
IL-1β	567 bp	ATGACCTGAGCACCTTCTTTC	TCTCTGGGTACAGCTCTCTTTA
IL-10	677 bp	GAACCAAGACCCAGACATCAA	CCAAGCCCAGAGACAAGATAAA
IL-6	427 bp	CAGCTATGAACTCCTTCTCCAC	CTGGCTTGTTCCTCACTACTC
IL-18	505 bp	CCAAGGAAATCGGCCTCTATT	GTCTTGAACACCTGACCTCTG
TNF-α	828 bp	ATCTACTCCCAGGTCCTCTTC	CCCGGTCTCCCAAATAAATACA
TGF-β	662 bp	GTGGAAACCCACAACGAAATC	GTGTCCAGGCTCCAAATGTA
β-actin	719 bp	GATCAAGATCATTGCTCCTCCTGAGCGCA	GTCTCAAGTCAGTGTACAGGTAAGCCCT

## Data Availability

The data that support the findings of this study are available from the corresponding author upon reasonable request.

## Supplementary Materials

The following supporting information can be downloaded at: https://www.mdpi.com/article/10.3390/biomedicines13020339/s1, Figure S1: Macrophages retained an M2 (pro-tumorigenic) or M1-like (pro-inflammatory) phenotype after 48 h of incubation with conditioning medium from untreated HT29 or *Lp*OC01-SN precured HT29 cells, followed by an additional 24 h incubation in normal medium.

## Abbreviations

The following abbreviations are used in this manuscript:

TAMs	Tumor-associated macrophages
CRC	Colorectal cancer
TME	Tumor microenvironment
IL	Interleukin
CM	Conditioning medium
TGF LPS	Tumor growth factor Lipopolysaccharide
ECM	Extracellular matrix
TNF	Tumor necrosis factor
ROS	Reactive oxygen species
PMA	Phorbol-12-Myristate-13-Acetate
COOH-NPs	Carboxy-functionalized nanoparticles
NM	Normal media
*Lp*OC01-SN	*Lactiplantibacillus plantarum* OC01 supernatant
CSF-1	Colony-stimulating factor-1
