# Supplementary figures and images for: Probiotic-Derived Metabolites from Lactiplantibacillus plantarum OC01 Reprogram Tumor-Associated Macrophages to an Inflammatory Anti-Tumoral Phenotype: Impact on Colorectal Cancer Cell Proliferation and Migration
Source: Biomedicines. 2025 Feb 3;13(2):339. doi: 10.3390/biomedicines13020339 (PMC11853712; doi:10.3390/biomedicines13020339)

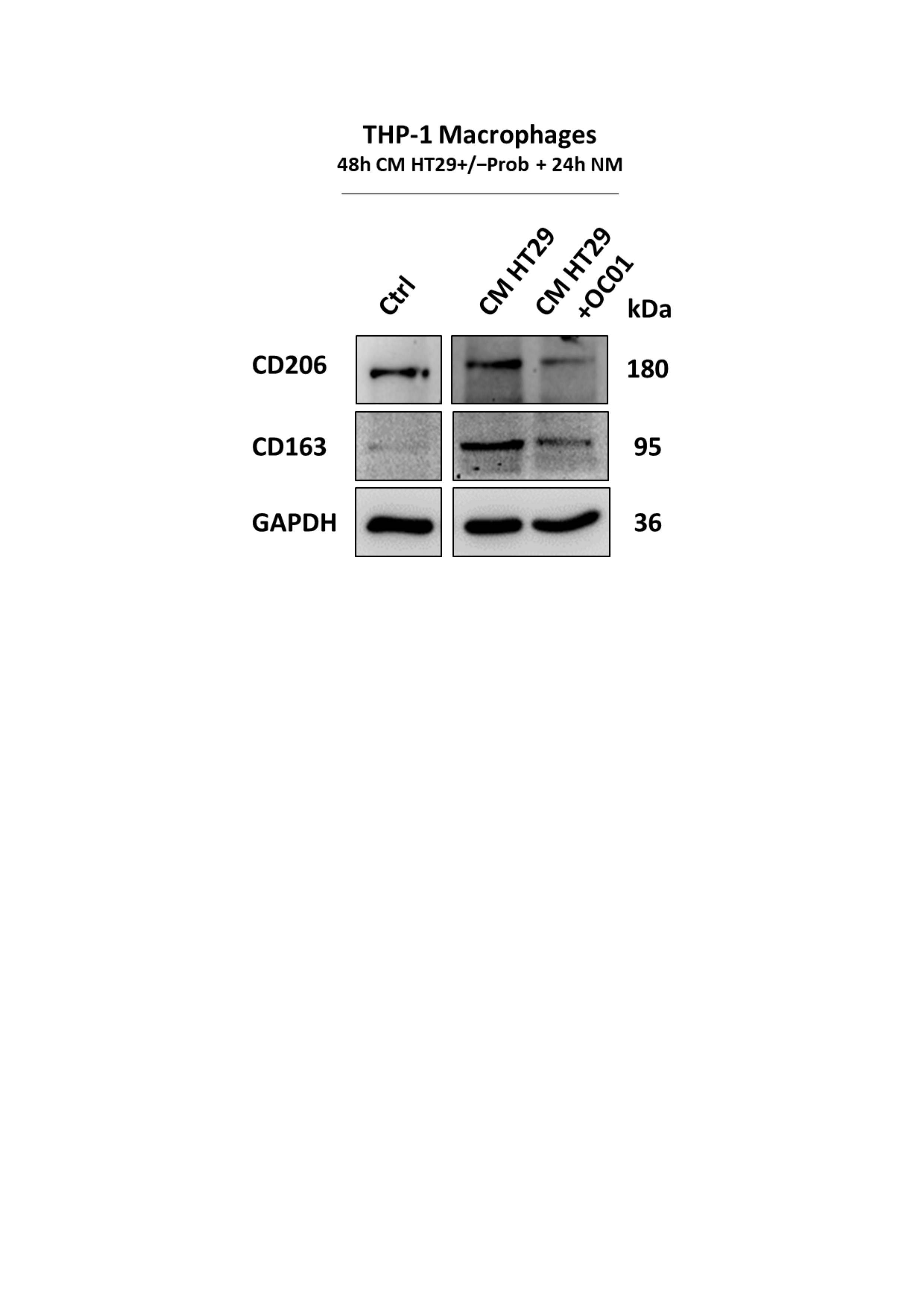

Supplement: Supplementary file 1 [file biomedicines-13-00339-s001.zip › biomedicines-3455168-supplementary.jpg]
